# Digit ratio (2D:4D) in relation to substance and computer use: a meta-analysis

**DOI:** 10.1007/s00702-019-02002-2

**Published:** 2019-04-27

**Authors:** Eva-Maria Siegmann, Polyxeni Bouna-Pyrrou, Bernd Lenz, Johannes Kornhuber

**Affiliations:** Department of Psychiatry and Psychotherapy, Friedrich-Alexander University Erlangen-Nürnberg (FAU), Schwabachanlage 6, 91054 Erlangen, Germany

**Keywords:** Prenatal androgen exposure, 2D:4D, Testosterone, Androgens, Brain organization, Substance use, Alcohol, Tobacco

## Abstract

Human studies have reported inconsistent associations between the length ratio of the second finger to the fourth finger (2D:4D), which is a proxy for prenatal androgen load, and substance or computer use in adolescents and adults. This meta-analysis quantifies the magnitude of this relationship and investigates the roles of sex, definition of caseness, different forms of addiction, the hand measured (right hand versus left hand), and other cohort characteristics. Univariate random-effects meta-analyses were performed, and moderators were tested with Bonferroni-corrected meta-regression analyses. The study included 18 independent samples with a total of 175,955 participants (96,316 males and 79,639 females). There was a significant difference in 2D:4D between the substance and computer-using subjects and the controls for the combined sample (Hedge’s *g* = − 0.178 [− 0.291; − 0.064]) and for males (Hedge’s *g* = − 0.260 [− 0.399; − 0.122]), but not for females. These effects were amplified when only analyzing studies that compared dependent versus non-dependent subjects (combined sample: *g* = − 0.325 [− 0.492; − 0.157]; males: *g* = − 0.427 [− 0.564; − 0.291]), but did not reach significance in the subgroup of studies examining other parameters of substance and computer use. When analyzing different forms of substance and computer use separately, alcohol intake and computer use revealed a significant difference in the standardized mean. Again, the effects were amplified when analyzing the subgroup of males and the subgroup of studies comparing dependent versus non-dependent subjects, with effect sizes showing Hedge’s *g* values as many as − 0.552 [− 0.785; − 0.319] (alcohol-dependent males). Thus, this meta-analysis confirms that lower 2D:4D is associated with substance and computer dependency. Further studies are encouraged to explore the link between intrauterine hormone environment and addiction risk.

**Electronic supplementary material:**

The online version of this article (10.1007/s00702-019-02002-2) contains supplementary material, which is available to authorized users.

## Introduction

Substance use and addictive behaviors cause great public health burdens, and available strategies to reduce these burdens are limited. To establish novel preventive options, improved knowledge is needed in regard to the mechanisms that result in unhealthy substance use and addictive behaviors. It has been suggested that prenatal organization of the brain as a result of sex hormones has permanent effects on adult behaviors and may represent a mechanism with preventive potential (Lenz et al. 2012, 2017).

Translational evidence indicates that intrauterine androgen priming organizes the brain with lasting structural and molecular alterations and influences substance use and addictive behaviors in adulthood. In female sheep, prenatal exposure to excess testosterone increases the number of tyrosine hydroxylase-immunoreactive cells in the ventral tegmental area (VTA) of adult sheep (Brown et al. 2015). The VTA is a part of the mesolimbic system, which is an area of the brain that is related to motivation and addictive behaviors. Another study on mice showed that prenatal modulation of androgen receptors changes the levels of the neurotransmitters dopamine, serotonin, and noradrenaline during adulthood. These changes occur in the prefrontal cortex, hypothalamus, and ventral striatum, and the effects depend on the neurotransmitter, brain region, and sex. Moreover, prenatal intervention sex specifically alters gene expression of brain-derived neurotrophic factor, opioid receptor mu 1, and period circadian clock 2 in the brain of adult mice (Huber et al. 2018). All these factors have been linked to addiction pathology in previous research. Accordingly, prenatal androgen receptor antagonism with flutamide also decreased adult alcohol intake among males and prenatal androgen receptor activation increased alcohol intake among females (Huber et al. 2018). Thus, multilevel evidence from animals indicates that the prenatal reinforcement of the androgen signal increases later substance use, which involves structural and molecular brain organization that are relevant to addiction pathology.

Ethical reasons and the long time period between the intrauterine window and adulthood in humans hamper direct investigation of the effects of prenatal androgens on adult behaviors. Thus, this research area mainly relies on proxies that develop in utero and that do not strongly change afterwards. It is said that the length ratio of the second finger to the fourth finger (2D:4D) is a somewhat suitable biomarker in that lower 2D:4D indicates higher prenatal androgen load (Cohen-Bendahan et al. 2005; Berenbaum et al. 2009; Manning et al. 2014) (see also “Discussion”). 2D:4D is established during the first trimester and does not strongly change afterwards (Malas et al. 2006). It is frequently used in studies that investigate the association between prenatal androgen exposure and postnatal phenotypes including diseases, mental disorders, and behaviors during adulthood.

Vehmas et al. (2006) and Manning and Fink (2011) conducted initial studies linking 2D:4D with substance use. The amount of consumption, however, does not indicate a dependency on substance or computer use and is not a part of the criteria for substance use disorder, internet gaming disorder, or dependence according to DSM-5 or ICD-10. Instead, these criteria include aspects such as a strong desire to take a substance or to use the computer, difficulties in termination, withdrawal symptoms, evidence of tolerance such that increased doses are required to achieve effects, and neglect of important social, occupational, or recreational activities.

We have published the first studies on 2D:4D in substance-bound addiction (alcohol: Kornhuber et al. 2011) and non-substance-bound addiction (addictive computer use: Kornhuber et al. 2013). In both studies, we found smaller 2D:4D values in people with addictions than in control subjects. Our replication study on 2D:4D and alcohol addiction showed lower 2D:4D in alcohol-dependent males, whereas there was no significant difference in females (Lenz et al. 2017). The study on computer use indicated that 2D:4D is associated with addictive computer use (Kornhuber et al. 2013).

Many studies have since been conducted on the association between 2D:4D, substance use, and addictive behaviors. Although most studies showed that lower 2D:4D was related to substance and computer use, others reported null results and opposite relationships. Moreover, potential confounders have not yet been systematically investigated in a specific meta-analysis (including sex, definition of caseness, study quality, procedure of measuring 2D:4D, and mean age).

Based on our observations, we derived the following hypotheses for this meta-analysis: (1) 2D:4D is smaller in subjects with substance and computer use compared to controls. (2) This effect is stronger in males than females. (3) This effect is stronger when comparing dependent versus non-dependent subjects according to dependence criteria (i.e., the method of caseness definition) than when examining other parameters of substance and computer use. Conclusively, we expect the strongest effect in male subjects when dichotomizing according to dependence criteria. Furthermore, the aim of this meta-analysis is to examine how the relationship varies across different forms of substance and computer use (alcohol, illegal drugs, tobacco, and addictive computer use), right-hand 2D:4D versus left-hand 2D:4D, difference between right-hand and left-hand 2D:4D (Dr-l), 2D:4D measurement via quantification with and without soft tissue deformation, and mean age.

## Methods

### Search strategy

A two-step literature search was performed using Google Scholar and PubMed. The search was conducted from inception until August 7, 2018, and included abstracts in English. The following search terms were combined in several ways: 2D:4D, 2nd to 4th digit ratio, second to fourth digit ratio, second-to-fourth finger length ratio, index and ring digit length ratio, addiction, substance use disorder, dependence, intoxication, drugs, nicotine, smoking, alcohol, cannabis, cannabinoid, amphetamine, opiate, morphine, heroin, cocaine, gambling, gaming, behavioral addiction, and internet addiction. In the second step, the reference lists of retrieved articles were searched manually. The abstracts of all these articles were screened using our selection criteria. Based on a full-text review, the remaining articles were checked for eligibility according to the PRISMA statement (Liberati et al. 2009).

### Study selection

We included case–control studies, cohort studies, and cross-sectional studies. Our eligibility criteria comprised the following: publication date ranging from 1983 to 2018, only studies published in English language, 2D:4D measured and reported in a continuous way, examining either the correlation of 2D:4D with any form of substance and computer use or addiction and/or the comparison of substance or computer-using people (cases) with controls. In case–control studies, the controls must not have been diagnosed with any form of addiction. For inclusion, a study had to report enough data to compute effect sizes. Data that were not directly reported were extracted indirectly from associated values or obtained by contacting the authors.

Studies were excluded if the following criteria were met: only abstracts or pilot data available, studies published in languages other than English, and animal studies. Furthermore, a study was excluded if 2D:4D was measured in a categorical way (e.g., the index finger being smaller than the ring finger and vice-versa) and if only partial correlations or *β*-coefficients derived from multiple regression models were available. The literature search was summarized according to the PRISMA guidelines (Moher et al. 2010).

### Data extraction

Data extraction was performed by two investigators (E-MS and PB-P) according to a previously defined coding protocol (Online Resource 1). Disagreement was resolved by discussion and compromise on the extracted values. The extractors’ values were averaged for quality assessment. All recorded variables can be found in the coding protocol (Online Resource 1).

We collected additional moderators as stated in the “Statistical analysis”. The risk of bias was assessed with either the Newcastle–Ottawa Scale for case–control studies (Wells et al. 2018) or an adaptation of the Newcastle–Ottawa Scale for cohort studies (Wells et al. 2018), which is specifically designed for cross-sectional studies. For this purpose, we borrowed the adaptation developed by Herzog et al. (2013) for their systematic review. This tool consists of three categories (selection, comparability, and exposure) with a total of 8 items (7 items in the case of the adaptation). A study can be awarded a maximum of one star for each item within the selection and exposure category and a maximum of two stars can be given for comparability. A higher overall quality sum score reflects greater study quality.

### Statistical analysis

#### Meta-analyses

For the main analysis, we estimated the standardized mean difference (Hedge’s *g*) in 2D:4D among substance or computer-using subjects and controls. Thus, correlative data were transformed into Hedge’s *g* using common transformation formulas (Borenstein et al. 2009, pp 45–49). Hedge’s *g* is an effect size that quantifies mean differences in a similar way to Cohen’s *d*, but it corrects the pooled standard deviation (Hedges 1981). The interpretation of Hedge’s *g* and Cohen’s *d* is comparable. The analysis was performed for both sexes combined and for men and women separately to detect sex-specific effects. We tested with fixed-effect models whether male and female meta-analysis estimates differed significantly.

Then, we tested for standardized mean differences in Dr-l in substance and computer-using subjects and non-dependent controls. Low Dr-l values have been associated with high prenatal testosterone load beyond 2D:4D (Manning 2002, pp 21–22). This analysis was feasible for all studies which reported means and standard deviations of an affected and a non-affected group. Dr-l was computed as the difference between the mean right-hand 2D:4D and mean left-hand 2D:4D, and related standard deviations were approximated by the pooled standard deviation of the right-hand 2D:4D and left-hand 2D:4D variances.

Furthermore, we tested whether males had a smaller 2D:4D than females and whether the right-hand 2D:4D is smaller than the left-hand 2D:4D among subjects in our analysis. Both are prominent findings and often replicated in 2D:4D research (Hönekopp and Watson 2010; Xu and Zheng 2015). All analyses were conducted using the metafor package (Viechtbauer 2010) within the open-source software environment R, version 3.4.2. (R Core Team 2018).

We performed univariate meta-analyses using restricted maximum likelihood estimation. The point estimate for each study was weighted by the inverse of its variance. Nonindependence among effect sizes was accounted for by aggregating. Heterogeneity among effect sizes within datasets was assessed using the *I*^2^ statistic. This statistic can be interpreted as the percentage of the total variability in a set of effect sizes due to between-study variability (Cochrane Training 2018). The Cochrane handbook proposes a tentative classification where an *I*^2^ of 0–40% might not be important, *I*^2^ of 30–60% indicates moderate heterogeneity, *I*^2^ of 50–90% indicates substantial heterogeneity, and *I*^2^ greater than 75% indicates considerable heterogeneity.

#### Meta-regressions and subgroup analyses concerning the main meta-analysis

To explain the residual heterogeneity and to understand the potential effect of contextual factors on the outcomes, we ran pre-specified meta-regression analyses for the following moderators: study quality, mean age, and procedure of measuring 2D:4D. The latter refers to whether 2D:4D was measured by multiple independent raters, multiple times by one rater, once by one rater, or by the participants themselves. Thus, the slope of the meta-regression line (*β* coefficient) indicates the strength of the association between the moderator and outcome.

The meta-regressions were Bonferroni-corrected for multiple testing. We performed pre-specified subgroup analyses to investigate the difference in the outcome measures between (1) the definition of caseness (studies comparing dependent with non-dependent subjects according to diagnostic criteria versus studies examining other parameters of substance and computer use); (2) the left hand and right hand; (3) the different addiction forms: alcohol, illegal drugs, tobacco, or addictive computer use (it was not possible to test for gambling separately since only one independent study reported relevant data); and (4) the different methods of measuring 2D:4D. The latter was dichotomized into “measurement without soft-tissue deformation” (comprising X-rays and direct measurement from the participants’ palm) and “measurement with soft-tissue deformation” (comprising photocopies and hand scans).

Concerning the definition of caseness, two subgroups were formed as follows. A study was assigned to the group of studies comparing dependent with non-dependent subjects when cases were identified according to ICD-10, DSM-IV, or DSM-5 criteria, as well as comparable questionnaires that allow for clear, diagnostic decisions, such as the Internet Addiction Test (Young 1998) and the Video Game Addiction Scale (CSAS-II) (Rehbein et al. 2010). The remaining studies were clustered into the group of “other studies” since they did not compare dependent with non-dependent subjects according to dependency criteria but studied other parameters of addictive behavior.^1^

#### Publication bias and sensitivity analyses

Publication bias and small study effects were assessed with the funnel function of R, which produced contour-enhanced funnel plots for the visual detection of asymmetries. In addition, the Egger regression test was used to detect asymmetry in the funnel plots (Egger et al. 1997). We considered analyses to be biased if the intercept differed from zero at *p* = 0.10, as the authors originally proposed (Egger et al. 1997). We evaluated the sensitivity of our analysis by comparing models with and without effect sizes, which we assume to be influential outliers (Viechtbauer and Cheung 2010). A study may be considered to be influential if at least one of the following is true: (1) the absolute DFFITS value is larger than 3√(*p*/(*k* – *p*)), where *p* is the number of model coefficients, and *k* the number of studies. (2) The lower tail area of a Chi-squared distribution with *p* degrees of freedom cut off by the Cook’s distance is larger than 50%. (3) The hat value is larger than 3(*p*/*k*). (4) Any DFBETAS value is larger than 1 (Viechtbauer and Cheung 2010).

*P* < 0.05 (two-sided) was considered statistically significant, except for the regression test for small study effects as stated above.

## Results

### Eligible studies

**Fig. 1:**
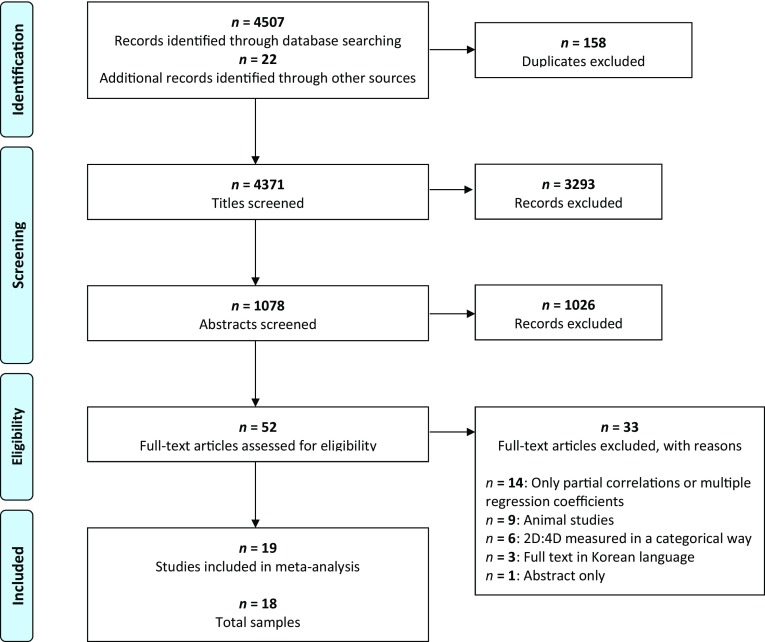
A PRISMA flow diagram illustrating the literature search

The literature search (Fig. 1) identified 19 partly dependent articles (Vehmas et al. 2006; Kornhuber et al. 2011, 2013; Manning and Fink 2011; Herschl et al. 2012; Borkowska and Pawlowski 2013; Romero-Martínez et al. 2013; Park et al. 2014; Hoskin and Ellis 2015; Han et al. 2016; Canan et al. 2017, 2018; Lenz et al. 2017, 2018a; Müller et al. 2017; Cicek et al. 2017; Hoskin 2017; Hobson 2018; Kim et al. 2018) and 18 independent samples. Two studies (Hoskin and Ellis 2015; Hoskin 2017) are based on the same collective and, therefore, count as one independent sample in our analysis. The evidence is from seven areas: German-speaking regions (Austria, Germany, and Switzerland), South Korea, Finland, Poland, Turkey, Spain, and the United States, along with one international online study (Manning and Fink 2011).

The characteristics of all included studies are detailed in Tables 1 and 2, and separated according to whether they reported data on standardized mean differences or correlative data, as well as male and female data. Some of the articles reported both case versus control data as well as correlations.

**Table 1:** Characteristics of studies reporting case–control data

First author, year of publication	Substance and computer-using subjects				Controls				
	Dichotomization according to dependence criteria								
	${n_{{\text{males}}}}$	2D:4D_males_ (M ± SD)	${n_{{\text{females}}}}$	2D:4D_females_ (M ± SD)	${n_{{\text{males}}}}$	2D:4D_males_ (M ± SD)	${n_{{\text{females}}}}$	2D:4D_females_ (M ± SD)	Subforms of addiction
Kornhuber et al. (2011)^a,e^	87	0.952 ± 0.031	44	0.967 ± 0.03	83	0.976 ± 0.029	102	0.983 ± 0.032	Alcohol
Kornhuber et al. (2011)^b,e^	87	0.949 ± 0.034	44	0.967 ± 0.031	83	0.967 ± 0.029	102	0.976 ± 0.03	Alcohol
Han et al. (2016)^a,e^	87	0.934 ± 0.026	NA	NA	52	0.956 ± 0.031	NA	NA	Alcohol
Han et al. (2016)^b,e^	87	0.942 ± 0.027	NA	NA	52	0.958 ± 0.029	NA	NA	Alcohol
Lenz et al. (2017)^a,c,e^	103	0.956 ± 0.026	79	0.972 ± 0.031	133	0.962 ± 0.028	105	0.963 ± 0.031	Alcohol
Lenz et al. (2017)^b,c,e^	103	0.956 ± 0.025	79	0.967 ± 0.031	133	0.97 ± 0.031	105	0.962 ± 0.033	Alcohol
Cicek et al. (2017)^a,d^	62	0.96 ± 0.03	NA	NA	50	0.98 ± 0.05	NA	NA	Illegal drugs
Canan et al. (2018)^a,d^	150	0.98 ± 0.03	NA	NA	266	0.99 ± 0.04	NA	NA	Illegal drugs
Canan et al. (2018)^b,d^	150	0.98 ± 0.03	NA	NA	266	0.99 ± 0.04	NA	NA	Illegal drugs
Kornhuber et al. (2013)^a,e^	27	0.967 ± 0.018	NA	NA	27	0.977 ± 0.025	NA	NA	Computer
Kornhuber et al. (2013)^b,e^	27	0.966 ± 0.02	NA	NA	27	0.982 ± 0.025	NA	NA	Computer
Canan et al. (2017)^a,d^	52	0.969 ± 0.028	50	1.017 ± 0.039	231	0.996 ± 0.033	319	1.001 ± 0.038	Computer
Canan et al. (2017)^b,d^	52	0.978 ± 0.034	50	0.998 ± 0.036	231	0.993 ± 0.346	319	0.996 ± 0.038	Computer
Kim et al. (2018)^a,d^	164	0.949 ± 0.03	49	0.981 ± 0.04	224	0.955 ± 0.03	216	0.985 ± 0.05	Computer

	Dichotomization not according to dependence criteria								
	*n*_males_	2D:4D_males_ (M ± SD)	${n_{{\text{females}}}}$	2D:4D_females_ (M ± SD)	${n_{{\text{males}}}}$	2D:4D_males_ (M ± SD)	${n_{{\text{females}}}}$	2D:4D_females_ (M ± SD)	Subforms of addiction
Romero-Martínez et al. (2013)^a,e^	74	0.95 ± 0.09	NA	NA	71	0.99 ± 0.04	NA	NA	Alcohol
Romero-Martínez et al. (2013)^b,e^	74	1 ± 0.05	NA	NA	71	0.99 ± 0.07	NA	NA	Alcohol
Lenz et al. (2018b)^a,c,d^	580	0.985 ± 0.035	530	0.99 ± 0.037	355	0.993 ± 0.035	459	0.992 ± 0.034	Alcohol
Lenz et al. (2018b)^b,c,d^	580	0.985 ± 0.036	530	0.988 ± 0.038	355	0.992 ± 0.035	459	0.991 ± 0.036	Alcohol
Borkowska et al. (2013)^a,d^	111	0.979 ± 0.03	117	1 ± 0.034	119	0.981 ± 0.029	108	0.996 ± 0.033	Nicotine
Borkowska et al. (2013)^b,d^	111	0.992 ± 0.035	117	1.006 ± 0.03	119	0.991 ± 0.031	108	0.996 ± 0.033	Nicotine
Park et al. (2014)^a,d^	69	0.953 ± 0.031	NA	NA	93	0.956 ± 0.038	NA	NA	Nicotine
Canan et al. (2018)^a,d^	77	0.991 ± 0.036	NA	NA	189	0.996 ± 0.044	NA	NA	Nicotine
Canan et al. (2018)^b,d^	77	0.986 ± 0.056	NA	NA	189	0.991 ± 0.041	NA	NA	Nicotine

^a^Right 2D:4D

^b^Left 2D:4D

^c^Authors contacted for this data

^d^No tissue deformation when measuring 2D:4D

^e^Tissue deformation when measuring 2D:4D

**Table 2:** Characteristics of studies reporting correlative data

First author, year of publication	Males		Females		Subforms of addiction
	*n*	*r*_p_	*n*	*r*_p_	
Vehmas et al. (2006)^a,d,e^	NA	NA	490	− 0.039	Alcohol
Manning et al. (2011)^a,e^	92,686	− 0.018	76,488	− 0.03	Alcohol
Manning et al. (2011)^b,e^	92,686	− 0.019	76,488	− 0.022	Alcohol
Lenz et al. (2018b)^a,d,e^	935	– 0.03	989	0	Alcohol
Lenz et al. (2018b)^b,d,e^	935	− 0.024	989	0	Alcohol
Lenz et al. (2018b)^a,d,e^	935	− 0.105	989	− 0.032	Alcohol
Lenz et al. (2018b)^b,d,e^	935	− 0.086	989	− 0.058	Alcohol
Herschl et al. (2012)^c,f^	60	0.3	NA	NA	Illegal drugs
Hoskin et al. (2015)^a,f,g^	190	− 0.186	255	− 0.21	Illegal drugs
Cicek et al. (2017)^a,e^	62	− 0.014	NA	NA	Illegal drugs
Hoskin (2017)^a,f,g^	190	− 0.186	255	− 0.193	Illegal drugs
Canan et al. (2018)^a,e,g^	150	− 0.129	NA	NA	Illegal drugs
Canan et al. (2018)^b,e,g^	150	− 0.164	NA	NA	Illegal drugs
Hobson (2018)^a,e^	36	− 0.36	90	0.03	Illegal drugs
Hobson (2018)^b,e^	36	− 0.51	90	0.01	Illegal drugs
Manning and Fink (2011)^a,e^	92,305	0.004	76,443	0.022	Nicotine
Manning and Fink (2011)^b,e^	92,305	0.01	76,443	0.019	Nicotine
Lenz et al. (2017)^a,f,g^	87	0.287	NA	NA	Nicotine
Lenz et al. (2017)^b,f,g^	87	0.142	NA	NA	Nicotine
Canan et al. (2017)^a,e^	283	− 0.353	369	0.011	Computer
Canan et al. (2017)^b,e^	283	− 0.192	369	− 0.033	Computer
Müller et al. (2017)^a,f,g^	74	− 0.199	138	0.052	Computer
Müller et al. (2017)^b,f,g^	74	0.01	138	− 0.021	Computer
Kim et al. (2018)^a,e^	388	− 0.139	265	0.035	Computer
Hoskin and Ellis (2015)^a,f,g^	190	− 0.296	255	− 0.197	Gambling
Hoskin (2017)^a,f,g^	190	− 0.296	255	− 0.197	Gambling

^a^Right 2D:4D

^b^Left 2D:4D

^c^Mean of right and left 2D:4D

^d^The authors contacted for these data

^e^No tissue deformation when measuring 2D:4D

^f^Tissue deformation when measuring 2D:4D

^g^Pearson *r* derived from Spearman *ρ*

### Main analysis: strongest effect in males and dependent subjects

In line with hypothesis 1, we found that 2D:4D was smaller in subjects with substance and computer use compared to controls with a Hedge’s *g* of – 0.178 (Table 3a).

**Table 3:** Meta-analysis results of the main analysis, the subgroup analysis of definition of caseness, and the subgroup analysis of addiction subforms (Hedge’s *g* [95% CI]; *k*)

**(a) Mean differences (Hedge’s** ***g*** **) in 2D:4D**	
Males and females	
**− 0.178 [− 0.291; − 0.064]**; *k* = 18	

Males	Females
**− 0.260 [− 0.399; − 0.122]**; *k* = 17	− 0.031 [− 0.147; 0.085]; *k* = 11

**(b) Mean differences (Hedge’s** ***g*** **) in 2D:4D according to definition of caseness**			
Males and females			
Dichotomization according to dependency criteria			
Yes		No	
**− 0.325 [− 0.492; − 0.157]**; *k* = 8		− 0.093 [− 0.202; 0.016]; *k* = 15	

Males Dichotomization according to dependency criteria		Females Dichotomization according to dependency criteria	
Yes	No	Yes	No
**− 0.427 [− 0.564; − 0.291]**; *k* = 8	− 0.157 [− 0.320; 0.006]; *k* = 14	0.011 [− 0.290; 0.311]; *k* = 4	− 0.037 [− 0.139; 0.066]; *k* = 9

**(c) Mean differences (Hedge’s *g***) **in 2D:4D according to addiction subforms**						
Males and females			Males and females Dichotomization according to dependency criteria			
			Yes		No	
Alcohol	**− 0.206 [− 0.392; − 0.019]**; *k* = 7		**− 0.411 [− 0.793; − 0.029]**; *k* = 3		− 0.051 [− 0.146; 0.044]; *k* = 4	
Illegal drugs	− 0.203 [− 0.527; 0.122]; *k* = 5		**− 0.317 [− 0.494; − 0.141]**; *k* = 2		− 0.157 [− 0.512; 0.198]; *k* = 5	
Nicotine	0.014 [− 0.116; 0.143]; *k* = 5		NA		0.014 [− 0.116; 0.143]; *k* = 5	
Computer use	**− 0.176 [− 0.297; − 0.055]**; *k* = 4		**− 0.172 [− 0.326; − 0.018]**; *k* = 3		**− 0.183 [− 0.333; − 0.034]**; *k* = 3	

	Males	Females	Males Dichotomization according to dependency criteria		Females Dichotomization according to dependency criteria	
			Yes	No	Yes	No
Alcohol	**− 0.317 [− 0.546; − 0.088]**; *k* = 6	− 0.029 [− 0.178; 0.119]; *k* = 5	**− 0.552 [− 0.785; − 0.319]**; *k* = 3	− 0.083 [− 0.222; 0.056]; *k* = 3	− 0.070 [− 0.707; 0.568]; *k* = 2	− 0.005 [− 0.084; 0.074]; *k* = 3
Illegal drugs	− 0.249 [− 0.682; 0.184]; *k* = 5	− 0.243 [− 0.622; 0.137] *k* = 2	**− 0.317 [− 0.494; − 0.141]**; *k* = 2	− 0.207 [− 0.666; 0.252]; *k* = 5	NA	–0.243 [− 0.622; 0.137]; *k* = 2
Nicotine	**− 0.037 [− 0.048; − 0.026]**; *k* = 5	0.058 [− 0.202; 0.318]; *k* = 2	NA	**− 0.037 [− 0.048; − 0.026]**; *k* = 5	NA	0.058 [− 0.202; 0.318]; *k* = 2
Computer use	**− 0.362 [− 0.543; − 0.180]**; *k* = 4	0.052 [− 0.082; 0.190]; *k* = 3	**− 0.350 [− 0.567; − 0.134]**; *k* = 3	**− 0.377 [− 0.606; − 0.147]**; *k* = 3	0.089 [− 0.223; 0.400]; *k* = 2	0.014 [− 0.114; 0.143]; *k* = 3

Two subgroups of studies (caseness) were defined depending on whether they dichotomized the sample according to dependency criteria. If yes, the studies compared dependent subjects identified according to ICD-10, DSM-IV, or DSM-5 criteria, as well as comparable questionnaires that allow for clear, diagnostic decisions with non-dependent subjects. If no, the studies investigated other parameters of substance and computer use

*k* number of studies included for the analysis,* NA* not applicable

Significant results are printed in bold font

When analyzing both sexes separately, 17 studies reported data on men, resulting in a Hedge’s *g* of – 0.260 (Table 3a) and 11 studies reported data on women, resulting in a Hedge’s *g* of – 0.031 (not significant, Table 3a). In line with hypothesis 2, these two effects differed significantly in that males showed a stronger effect compared to females (Test of moderators, QM = 6.174, *p* = 0.013).

Table 3b shows the results concerning the separation of our data in studies comparing dependent with non-dependent subjects according to dependency criteria and “other studies” (i.e., studies examining parameters of substance and computer use other than dependency criteria). We found a significant standardized mean difference for dependent versus non-dependent subjects, while the group of “other studies” revealed no significant effect. The same holds true for the male subsample, for which we found a Hedge’s *g* of – 0.427 for studies comparing dependent with non-dependent subjects according to dependency criteria and no significant effect for “other studies”.

Among women, no effect reached significance. The effect sizes of studies comparing dependent with non-dependent subjects according to dependency criteria and “other studies” differed significantly in the combined sample (QM = 5.120, *p* = 0.024) and in males (QM = 6.249, *p* = 0.012), but not in females (QM = 0.085, *p* = 0.771). For the combined dataset and the male subsample, results are in line with hypothesis 3: the effects are stronger when defining caseness by dichotomizing according to dependence criteria than when examining other parameters of substance and computer use.

Conclusively, we found the strongest effect in male-dependent subjects with a Hedge’s *g* of – 0.427.

Concerning Dr-l, none of the three meta-analyses of standardized mean differences revealed any significant association with substance or computer use (combined: *g* = − 0.066 [95% CI − 0.218; 0.085],^2^*k* = 10; men: *g* = − 0.075 [95% CI − 0.231; 0.082], *k* = 10; women: *g* = 0.009 [95% CI − 0.026; 0.007], *k* = 6).

### Analysis of addiction subforms: strongest effects in alcohol dependency

**Fig. 2:**
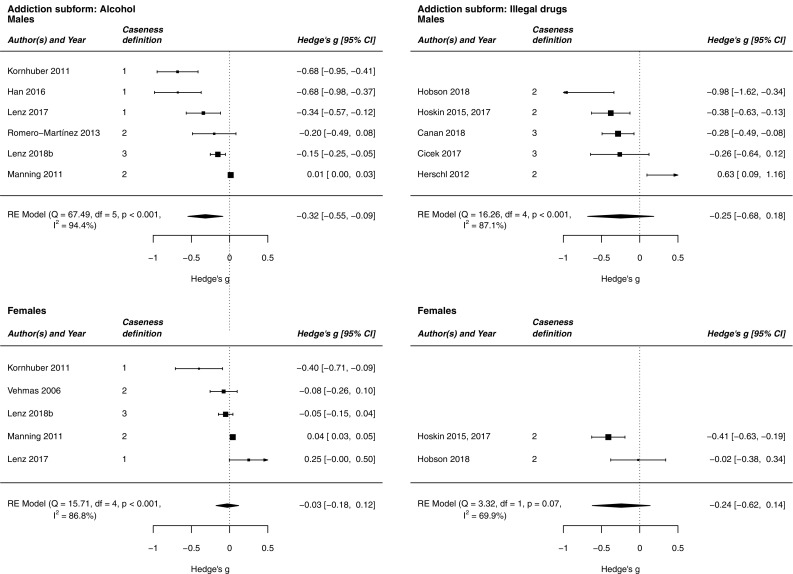
Forest plots showing the meta-analysis estimate of 2D:4D among alcohol and illegal drug-using subjects and controls. Caseness definition: the studies were separated in two subgroups depending on whether they dichotomized the sample according to dependency criteria. 1, The studies compared dependent subjects identified according to ICD-10, DSM-IV, or DSM-5 criteria, as well as comparable questionnaires that allow for clear, diagnostic decisions with non-dependent subjects. 2, The studies investigated other parameters of substance and computer use. 3, The studies used both mentioned caseness definitions

**Fig. 3:**
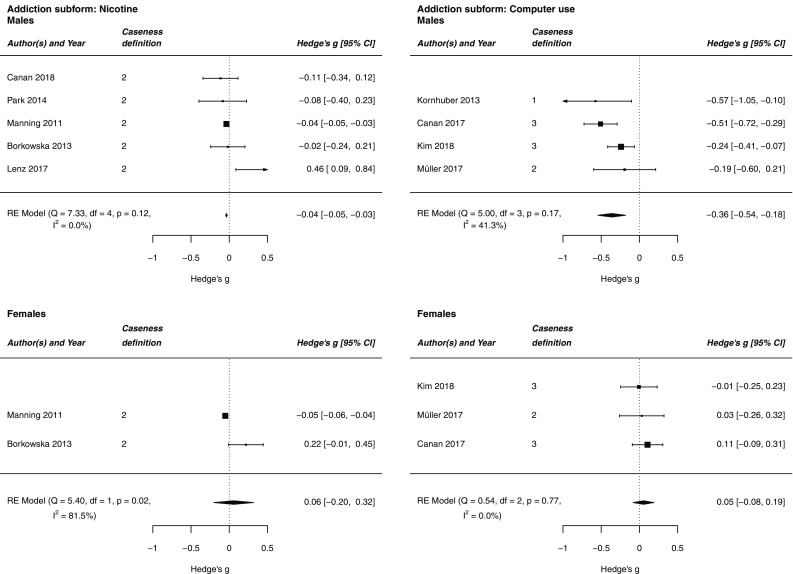
Forest plots showing the meta-analysis estimate of 2D:4D among tobacco smoking and computer-using subjects and controls. Caseness definition: the studies were separated in two subgroups depending on whether they dichotomized the sample according to dependency criteria. 1, The studies compared dependent subjects identified according to ICD-10, DSM-IV, or DSM-5 criteria, as well as comparable questionnaires that allow for clear, diagnostic decisions with non-dependent subjects. 2, The studies investigated other parameters of substance and computer use. 3, The studies used both mentioned caseness definitions

Table 3c and Figs. 2, 3 show the results of the separate analysis of different subforms of addiction. When analyzing the combined dataset, we found a significant standardized mean difference in alcohol intake (*g* = − 0.206) and addictive computer use (*g* = − 0.176), but not in illegal drug intake and smoking. The effects were amplified when analyzing the male subsample separately: alcohol intake (*g* = − 0.317), smoking (*g* = − 0.037), and addictive computer use (*g* = − 0.362) revealed a significant effect, while illegal drug intake did not. In the female subsample, none of the effects were significant.

With respect to alcohol and illegal drug intake, the effects were further amplified when separating according to studies comparing dependent with non-dependent subjects according to dependency criteria. The strongest effects were observed for alcohol-dependent subjects in general (*g* = − 0.411) and alcohol-dependent males (*g* = − 0.552). Concerning addictive computer use, there was no difference between studies comparing dependent with non-dependent subjects according to dependency criteria and “other studies”, i.e., studies examining other parameters of substance and computer use: both effects were significant in the combined sample and in males. Thus, the definition of caseness according to dependence criteria is not as relevant to the 2D:4D-computer use association as it is to the 2D:4D-substance use association. Due to a lack of data, it was not possible to test for differences between dependent smokers and smoking in the subgroup of “other studies”.

### Further subgroup analyses and meta-regressions

The subgroup analysis of the left hand only versus the right hand only revealed no significant differences for the combined sample, men, or women. Similarly, effect sizes did not differ between methods of measuring 2D:4D with soft tissue deformation and without soft tissue deformation for the combined sample, men, or women (data not shown).

None of the meta-regression analyses concerning study quality (*β* = − 0.029, *p* =  0.598), mean age (*β* = − 0.002, *p* = 0.812), and the procedure of measuring 2D:4D (*β* = 0.023, *p* = 0.662) showed any significant effects with a Bonferroni-corrected threshold of *p* = 0.017.

### Sensitivity analyses and small study effects

The Egger test and funnel plots showed no evidence of small study effects for the combined dataset, men only, or women only. Sensitivity analyses revealed no influential outliers regarding the meta-analyses of the combined dataset and the male subsample. Thus, our data concerning these datasets are rather robust. With respect to the meta-analysis of women, we found that one effect size [the combined value of Hoskin and Ellis (2015) and Hoskin (2017)] had a Cook’s distance and studentized residuals that were beyond our permitted range. We performed a second meta-analysis after excluding this influential effect size, which resulted in extreme reduction in heterogeneity (*I*^2^ = 0.02% compared to *I*^2^ = 84.48% in the original analysis), as well as a decrease in the outcome measure, although the result was still not significant (*g* = − 0.006 [95% CI − 0.017; 0.006] compared to *g* = − 0.031 [95% CI − 0.147; 0.085] in the original analysis).

### Replication of prominent 2D:4D findings

The meta-analysis revealed lower 2D:4D in males than females with Hedge’s *g* = 0.352 [95% CI 0.194; 0.510], hence replicating this prominent finding in our sample. When examining whether 2D:4D of the right hand was generally lower than 2D:4D of the left hand, we found no significant difference for the combined sample (*g* = − 0.006 [95% CI − 0.077; 0.066], *k* = 12), for men (*g* = − 0.052 [95% CI − 0.144; 0.039], *k* = 12), or for women (*g* = 0.065 [95% CI − 0.021; 0.151], *k* = 8). Thus, the replication of right-hand 2D:4D < left-hand 2D:4D was not possible in our sample.

## Discussion

In agreement with our first hypothesis, this meta-analysis revealed a significant association of 2D:4D with substance and computer use in that substance and computer use was related to a smaller 2D:4D. In the sex-separated analysis, the association was stronger in males than females, which confirms our second hypothesis that males show a more pronounced effect. The male effect size showed a Hedge’s *g* of – 0.260, which is in the small range according to Cohen’s (1988) guidelines. When analyzing only studies that dichotomized according to dependence criteria to identify cases, the effects were amplified: the mean difference in 2D:4D of studies comparing dependent with non-dependent subjects according to dependency criteria was stronger than the mean difference in 2D:4D of other studies that did not define caseness by dichotomizing according to dependence criteria. This result is in line with our third hypothesis. The strongest effect was found in the subsample of male-dependent subjects with a Hedge’s *g* of – 0.427. This effect is in the medium range according to a recent guideline for effect size, which revised Cohen’s classification (Gignac and Szodorai 2016). In contrast, there is no significant effect in the results of the subgroup of “other studies” (i.e., studies that did not directly compare dependent subjects with controls).

This pattern re-emerges when analyzing the different subforms of addiction separately. Alcohol intake, smoking, and addictive computer use, but not illegal drug intake, are associated with low 2D:4D in males but not in females. The male effect sizes are more pronounced than the overall effects for both sexes combined, but all significant effects are in the small range. There were almost no significant results in the subgroup of “other studies”, but studies comparing dependent with non-dependent subjects according to dependency criteria revealed effect sizes that were small (illegal drug dependency: *g* = − 0.317) to medium (alcohol dependency: *g* = − 0.411) according to Gignac and Szodorai (2016). The strongest effect can be found in male alcohol-dependent subjects (*g* = − 0.552).

Alcohol and illicit drug use disorders are approximately two times more common in men than in women (Merikangas and McClair 2012), while the difference between men and women in smoking is not as pronounced in high-income countries (World Health Organization 2018). The results of the sex-separated meta-analyses in subforms of addictions, therefore, match the sex differences in addictive behaviors: addiction subforms that are considerably more frequent in men were associated with significantly lower 2D:4D values in males in our analysis. The almost negligible association between 2D:4D and smoking found here (*g* = − 0.037) also fits to the results of the recently published largest ever genome-wide association study (GWAS) on 2D:4D which showed no relationship between the genetics of smoking and the genetics of 2D:4D (Warrington et al. 2018). Addictive computer use appears to be exceptional since we found significant effects in both studies comparing dependent with non-dependent subjects according to dependency criteria and in the subgroup of “other studies”, i.e., studies examining parameters of substance and computer use other than dependency criteria. This is possibly due to the fact that most of the diagnostic and correlative data regarding addictive computer use were assessed via the Internet Addiction Test (Young 1998) and are thus inter-related.

In summary, the strongest effects were found in males and when dichotomizing according to dependence criteria. The more heterogeneous the studies, the smaller the observed effects are. We conclude that 2D:4D is associated with dependence criteria rather than with other parameters of addiction, such as the amount of substance intake or time spent using the computer.

Like the sex difference shown in this meta-analysis, previous meta-analyses have observed lower 2D:4D in men than women, but also in the right hand compared to the left (Hönekopp and Watson 2010; Xu and Zheng 2015). In our analysis, however, we did not detect differences between 2D:4D of the right and left hands. This discrepancy compared to the findings reported by Hönekopp and Watson (2010) and Xu and Zheng (2015) is possibly a result of our screening of available literature for studies treating substance and computer use, whereas the other studies examined a larger and relatively unscreened cohort.

We also did not find a significant association of Dr-l with substance and computer use, which suggests that Dr-l is not as suitable as 2D:4D to indicate relations with substance or computer use. Other studies also report differences between measurements with and without soft tissue deformation (Hönekopp and Watson 2010; Xu and Zheng 2015; Ribeiro et al. 2016). However, it is unclear whether the measurement method influences the strength of the relationship between 2D:4D and target traits (Ribeiro et al. 2016). Our analysis did not confirm such an influence: the outcome measure did not differ between measurement methods with and without soft tissue deformation.

There is widely expressed criticism of the use of 2D:4D as a biomarker of prenatal testosterone (e.g., Voracek 2014; van Hemmen et al. 2017; Yeung and Tse 2017). For example, there is no X-linked inheritance in either classical genetic studies (Ramesh and Murty 1977; Voracek and Dressler 2009) or in GWAS (Warrington et al. 2018), although the androgen receptor is located on the *X* chromosome. Two meta-analyses show a lack of correlation between the effectiveness of androgen receptors (genetic CAG and GGC repeat length polymorphism) and 2D:4D (Hönekopp 2013; Voracek 2014). The largest GWAS on 2D:4D so far only shows a slight positive influence of CAG repeats in women, but not in men (Warrington et al. 2018). 2D:4D may thus be determined by not only prenatal testosterone, but also other factors, such as prenatal corticosterone (Lilley et al. 2010), prenatal stress (Lenz et al. 2017, 2018b), and genetic factors (Ramesh and Murty 1977; Paul et al. 2006; Voracek and Dressler 2007, 2009; Warrington et al. 2018). Future studies should define the factors influencing 2D:4D in addition to testosterone. This might also shed light on the pathophysiology of the predisposition of substance use and addictive behavior.

### Strengths and limitations

We have presented the first meta-analysis of the relationship between 2D:4D and substance and computer use. We have shown a consistent negative relationship with small to medium effect sizes between 2D:4D and addictive behavior in men, which was less clear in women. Many meta-analyses of 2D:4D in other fields show only small effect sizes between 2D:4D and variables of interest. Our meta-analysis is different and provides results in the range of the most pronounced meta-analytic 2D:4D associations: the highest effect sizes were previously found in regard to athletic prowess with correlations of up to *r* = − 0.26 (Hönekopp and Schuster 2010), in regard to autism spectrum disorders with mean differences of up to *d* = − 0.43 (Teatero and Netley 2013) and *d* = − 0.58 (Hönekopp 2012), and in regard to sexual orientation (*g* = − 0.55; − 0.44) (Grimbos et al. 2010).

The studies summarized in this meta-analysis come from different working groups, countries, continents, and ethnic groups. The analysis was conducted by entirely following the PRISMA statement (Liberati et al. 2009; Moher et al. 2010) and assessed study quality according to the Newcastle–Ottawa Quality Assessment Scale for case–control and cohort studies (Wells et al. 2018). This guarantees a standardized procedure for our analysis. In sensitivity analyses where one influential study was removed at a time from the models, the effects did not change appreciably. This suggests that the evidence does not depend on single studies and is reasonably robust. In our view, this underscores the value of 2D:4D as a research tool and also as a possible clinical tool. Our study thus contributes to the growing evidence for associations of 2D:4D with variables of interest as in meta-analyses for athletic prowess (Hönekopp and Schuster 2010), autism spectrum disorder (Hönekopp 2012), and sexual orientation in women (Grimbos et al. 2010).

There are some limitations to this study. First, the low number of studies allows only up to three conditions to be tested at a time (Table 3c: sex, definition of caseness, and addiction subform). Second, the high levels of heterogeneity could result in slightly overestimated effect sizes and could not always be explained by the pre-specified moderator analyses. Nevertheless, there are possible explanations for the partly high *I*^2^ statistic: we incorporated data from seven different regions and countries and one large international online study, we included a variety of subforms of addiction, and substance and computer use was assessed via various instruments that are not always perfectly comparable. We also accepted the possibility of high and significant heterogeneity a priori to integrate a large amount of data and to provide a broad overview of the existing evidence. Third, the study design of case–control studies entails some methodological problems (e.g., control self-selection resulting in information and selection bias), which can inflate the case–control difference and impede the generalizability of results. Fourth, compared to men, associations between substance and computer use and 2D:4D have been studied less frequently in women. This is probably due to the lower prevalence of addictive behaviors among women than men.

## Conclusions

Lower 2D:4D was significantly related to substance and computer use. This association was more pronounced in males than females and, for substance use, in studies comparing dependent with non-dependent subjects according to dependency criteria than in studies that did not dichotomize according to these criteria. The strongest standardized mean difference was found in male-dependent subjects, especially in male alcohol-dependent individuals. Further studies on 2D:4D, substance and computer use are desirable and should more often include women. Moreover, we suggest using clear dependence criteria instead of other dependence-related parameters, especially in regard to smoking. According to the results of the meta-analysis, no recommendation can be made as to whether future 2D:4D examinations should be performed using methods with or without soft tissue deformation. Future studies should define the role of 2D:4D alone or in combination with other markers in the prediction and prevention of substance and computer use.

## Electronic supplementary material

Below is the link to the electronic supplementary material.

Supplementary material 1 (DOCX 30 KB)

## Footnotes

1 Reasons why studies are clustered into the group of “other studies”: (1) reported correlative data with duration of disorder; (2) reported correlative data with lifetime substance use behavior; (3) reported correlative data with addiction test scores; (4) reported correlative data with intensity of addiction; (5) reported correlative data with amount of consumption (per week or per day); (6) cases were not defined according to ICD-10, DSM-IV, or DSM-5 criteria or following comparable questionnaires.

2 *k* = number of included studies.
