# Supplementary material for: Digit ratio (2D:4D) in relation to substance and computer use: a meta-analysis
Source: J Neural Transm (Vienna). 2019 Apr 27;126(5):623–36. doi: 10.1007/s00702-019-02002-2 (PMC6499759; doi:10.1007/s00702-019-02002-2)
Supplement: Supplementary file 1 — Supplementary material 1 (DOCX 30 KB) [file 702_2019_2002_MOESM1_ESM.docx]

Online Resource 1

Referring to article:

**Digit ratio (2D:4D) relates to substance and computer use: A meta-analysis**

Eva-Maria Siegmann, Polyxeni Bouna-Pyrrou, Bernd Lenz^1^, Johannes Kornhuber^1^*

Department of Psychiatry and Psychotherapy, Friedrich-Alexander University Erlangen-Nürnberg (FAU), Germany.

^1^Contributed equally

*Corresponding author at: Department of Psychiatry and Psychotherapy, Friedrich-Alexander University Erlangen-Nürnberg (FAU), Schwabachanlage 6, D-91054 Erlangen, Germany, Phone: +49 9131 85-34160, Fax: +49 9131 85-34862, E-Mail: johannes.kornhuber@uk-erlangen.de

**Coding protocol**

*2D:4D and addiction*

**Inclusion criteria:**

1. Year of publication: 1983-2018
2. Publication language: English
3. Digit ratio was measured as continuous measure
4. The correlation of digit ratio and any form of substance and computer use was examined or cases (addicted persons) and controls were compared concerning their digit ratio or both
5. In case-control studies, controls were not diagnosed with any form of addiction
6. Effect sizes or associated data to compute effect sizes are reported

**Exclusion criteria:**

1. Abstracts or pilot data
2. Publication language other than English
3. Animal studies
4. Digit ratio was measured as a categorical measure
5. Only partial correlations or β-coefficients from multiple regression models are reported

**Coding procedure:**

1. One line represents one effect size
2. If effect sizes are reported separately for the whole sample and for subgroups, only information concerning the whole sample will be extracted. If information is only reported for subgroups, every subgroup will be treated as distinct sample (variable sno).
3. If there are multiple effect sizes within one sample concerning different outcome variables (e.g. different ways of measuring 2D:4D), every effect size will be reported in its own line. These effect sizes are coded as dependent by allocating the same number for the variable sno.
4. No computations should be carried out while coding. Information is extracted directly without conversions.

| **Variable** | **Description** | **Code** | **Example** |
| --- | --- | --- | --- |
| ***General characteristics*** | | | |
| study | Study name comprising lead author and year of publication. | Free specification | Manning2000  Manning2000a |
| pubyear | Year of publication | Range: [1993, 2018] | 2007 |
| incl | Effect size cannot be included in statistical analysis | 0 = exclude  1 = include | 1 |
| sid | Consecutive number for every publication | Range: [1, ∞] | 1 |
| sno | Consecutive number for every sample | Range: [1, ∞] | 1 |
| colyear | Year of conduct | Range: [1993, 2018] | 2007 |
| cntry | Country of conduct.  If not reported, extract affiliation of lead author as ISO-CODE 2:  <http://en.wikipedia.org/wiki/ISO_3166-1_alpha-2>  or „XX“ describing samples with participants originating from different  countries | Free specification | DE |
| pubtype | Publication type | 1 = Peer-reviewed Journal  2 = Book  3 = Thesis (Master / PhD)  4 = Poster  5 = Other | 1 |

| **Variable** | **Description** | | **Code** | | **Example** |
| --- | --- | --- | --- | --- | --- |
| ***Sample***  ***(1=female; 2=male)*** | | | | | |
| n | | Sample size *N* | | Range: [2, ∞] | 100 |
| n1 | | Sample size of female sample | | Range: [2, ∞] | 100 |
| n2 | | Sample size of male sample | | Range: [2, ∞] | 100 |
| samtype | | Description of sample (coded) | | 1 = children  2 = students  (undergraduates, college)  3 = Adults, mixed  sample | 2 |
| age1 | | Mean age (in years) of female sample | | Range: [0, ∞] | 16.86 |
| age2 | | Mean age (in years) of male sample | | Range: [0, ∞] | 16.86 |
| 2d4d_m1 | | Mean 2D:4D of female sample | | Range: [0, ∞] | 0.9 |
| 2d4d_sd1 | | Standard deviation for 2D:4D of female sample | | Range: [0, ∞] | 0.1 |
| 2d4d_m2 | | Mean 2D:4D of male sample | | Range: [0, ∞] | 0.9 |
| 2d4d_sd2 | | Standard deviation for 2D:4D of male sample | | Range: [0, ∞] | 0.1 |
| trait_m1 | | Mean of substance or computer use of female sample | | Range: [-∞, ∞] | 5 |
| trait_sd1 | | Standard deviation of substance or computer use of female sample | | Range: [0, ∞] | 0.5 |
| trait_m2 | | Mean of substance or computer use of male sample | | Range: [-∞, ∞] | 5 |
| trait_sd2 | | Standard deviation of substance or computer use of male sample | | Range: [0, ∞] | 0.5 |

| **Variable** | **Description** | **Code** | | **Example** |
| --- | --- | --- | --- | --- |
| ***Effect sizes: correlation coefficients***  ***(1=female; 2=male)*** | | | | |
| r1 | Pearson’s correlation coefficient r for female sample | | Range: [-1, 1] | 0.5 |
| tau1 | Kendall’s τ for female sample | | Range: [-1, 1] | 0.5 |
| rho1 | Spearman’s rank correlation coefficient ρ for female sample | | Range: [-1, 1] | 0.5 |
| beta1 | Standardized regression coefficient (β) for simple linear regression for female sample | | Range: [-1, 1] | 0.5 |
| zr1 | Fisher’s r-to-z-transformed correlation coefficient for female sample | | Range: [-∞, ∞] | 1 |
| r2 | Pearson’s correlation coefficient r for male sample | | Range: [-1, 1] | 0.5 |
| tau2 | Kendall’s τ for male sample | | Range: [-1, 1] | 0.5 |
| rho2 | Spearman’s rank correlation coefficient ρ for male sample | | Range: [-1, 1] | 0.5 |
| beta2 | Standardized regression coefficient (β) for simple linear regression for male sample | | Range: [-1, 1] | 0.5 |
| zr2 | Fisher’s r-to-z-transformed correlation coefficient for male sample | | Range: [-∞, ∞] | 1 |

| **Variable** | **Description** | **Code** | | **Example** |
| --- | --- | --- | --- | --- |
| ***Effect sizes: Cohen’s d***  ***(1=female; 2=male)*** | | | | |
| n1_eg | Dependent group / cases: Sample size of female sample | | Range: [2, ∞] | 100 |
| n2_eg | Dependent group / cases: Sample size of male sample | | Range: [2, ∞] | 100 |
| n1_cg | Control group: Sample size of female sample | | Range: [2, ∞] | 100 |
| n2_cg | Control group: Sample size of male sample | | Range: [2, ∞] | 100 |
| m1_eg | Dependent group / cases: Female mean 2D:4D | | Range: [0, ∞] | 0.9 |
| sd1_eg | Dependent group / cases: Female standard deviation of 2D:4D | | Range: [0, ∞] | 0.1 |
| m2_eg | Dependent group / cases: Male mean 2D:4D | | Range: [0, ∞] | 0.9 |
| sd2_eg | Dependent group / cases: Male standard deviation of 2D:4D | | Range: [0, ∞] | 0.1 |
| m1_cg | Control group: Female mean 2D:4D | | Range: [0, ∞] | 0.9 |
| sd1_cg | Control group: Female standard deviation of 2D:4D | | Range: [0, ∞] | 0.1 |
| m2_cg | Control group: Male mean 2D:4D | | Range: [0, ∞] | 0.9 |
| sd2_cg | Control group: Male standard deviation of 2D:4D | | Range: [0, ∞] | 0.1 |

| **Variable** | **Description** | **Code** | | **Example** |
| --- | --- | --- | --- | --- |
| ***Additional information*** | | | | |
| qual_d1 | Category Selection   - Is the case definition adequate? - Representativeness of cases - Selection of controls - Definition of controls | | 0 = 0 Stars  1 = 1 Star  2 = 2 Stars  3 = 3 Stars  4 = 4 Stars | 1 |
| qual_d2 | Category Comparability: Comparability of cases and controls on the basis of the design or analysis   - Study controls for addiction - Study controls for any additional factor | | 0 = 0 Stars  1 = 1 Star  2 = 2 Stars | 1 |
| qual_d3 | Category Exposure   - Ascertainment of exposure - Same method of ascertainment for cases and controls - Non-response rate | | 0 = 0 Stars  1 = 1 Star  2 = 2 Stars  3 = 3 Stars | 1 |
| qual_r1 | Category Selection   - Representativeness of the sample - Sample size - Ascertainment of exposure - Non-respondents | | 0 = 0 Stars  1 = 1 Star  2 = 2 Stars  3 = 3 Stars  4 = 4 Stars | 1 |
| qual_r2 | Category Comparability: Confounding factors are controlled   - The study controls for the most important factor (diseases influencing 2D:4D or addiction) - The study controls for any additional factor | | 0 = 0 Stars  1 = 1 Star  2 = 2 Stars | 1 |
| qual_r3 | Category Outcome:   - Assessment of Outcome - The statistical test used to analyze the data is clearly described and appropriate | | 0 = 0 Stars  1 = 1 Star  2 = 2 Stars | 1 |
| **Variable** | **Description** | **Code** | | **Example** |
| ***Additional information*** | | | | |
| method | Method of measuring 2D:4D | | 1 = x-ray (without tissue deformation)  2 = photocopy or scans (with tissue deformation)  3 = directly from subjects’ hand (without tissue deformation) | 1 |
| rater | Who measured 2D:4D? How often was 2D:4D measured? | | 1 = multiple independent raters  2 = one rater performing multiple measurements  3 = one rater performing one measurement  4 = self-measurement by participants | 1 |
| hand | Subjects’ hand used to compute 2D:4D | | 1 = right hand only  2 = left hand only  3 = mean of both hands | 1 |
| addict | The form of addiction measured | | 1 = alcohol  2 = nicotine  3 = addictive computer use  4 = illegal drugs  5 = gambling | 1 |
